# Supplementary material for: Developmental Stages-Specific Response of Anise Plants to Laser-Induced Growth, Nutrients Accumulation, and Essential Oil Metabolism
Source: Plants (Basel). 2021 Nov 26;10(12):2591. doi: 10.3390/plants10122591 (PMC8708645; doi:10.3390/plants10122591)
Supplement: Supplementary file 1 [file plants-10-02591-s001.zip › plants-1432561-supplementary.pdf]

Table S1. Retention index essential oil measured by GC mass and retention time of phenolics and flavonoids profile measured by HPLC in fruits, sprouts and mature anise.

Essential oils	Retention Indices (RI)	fruits	Peak area	Control sprouts	Peak area	laser-treated sprouts	Peak area	Control Mature	Peak area	laser-treated Mature	Peak area
α-pinene	931	0.02	26.50	0.02	26.50	0.05	66.2627	0.05	66.26	0.04	53.01
Sabinene	971	0.26	344.5	0.46	609.61	0.34	450.58	0.2	265.05	0.25	331.31
Myrcene	981	0.13	172.2	0.15	198.78	0.45	596.36	0.39	516.84	0.34	450.58
Fenchone	1098	3.3	4373.3	4.38	5804.61	4.79	6347.96	3.61	4784.16	5.09	6745.54
p-cymene	1074	0.47	622.8	0.61	808.40	0.62	821.65	0.48	636.12	0.59	781.89
o-isoeugenol	1444	3.9	5168.4	2.97	3936.00	4.47	5923.88	3.47	4598.63	4.17	5526.30
1,8-cineole	1024	1.24	1643.3	1.41	1868.60	1.84	2438.46	1.61	2133.65	1.72	2279.43
Cis-β-ocimene	1035	0.26	344.5	0.33	437.33	0.51	675.87	0.4	530.10	0.47	622.86
Aα-phellandrene	1004	0	0	0	0	0	0	0.14	185.53	0.012	15.90
Methyl chavicol	1201	0.18	238.5	0.2	265.05	0.39	516.84	0.16	212.04	0.23	304.80
Endo-fenchyl acetate	1224	0	0	0.001	1.32	0.002	2.65	0	0	0	0
p-anisaldehyde	1253	0.07	92.7	0.08	106.02	0.06	79.51	0.05	66.26	0.08	106.02
Limonene	1027	0.12	159.0	0.18	238.54	0.35	463.83	0.23	304.80	0.3	397.57
Stearic acid	2157	0.03	39.7	0.03	39.75	0.03	39.75	0.03	39.75	0.03	39.75
2-oleoylglycerol	2153	0.19	251.7	0.29	384.32	0.44	583.11	0.29	384.32	0.41	543.35
γ-himachalene	1501	0.1	132.5	0.11	145.77	0.11	145.77	0.1	132.52	0.12	159.03
Trans-pseudoisoeugenyl	1802	0.13	172.2	0.15	198.78	0.16	212.04	0.14	185.53	0.17	225.29
Trans-anethole	1209	44	58311.176	49.9	66249.44	75.1	99619.3	66.4	87996.8	70.5	93430.40

Retention index (RI) on a non-polar column

Table S2. Retention time of phenolics and flavonoids profiles measured by HPLC in fruits, sprouts and mature anise.

Phenolic acids/ Flavonoids	Retention Time (min)	fruits	Peak area	Control sprouts	Peak area	laser-treated sprouts	Peak area	Control Mature	Peak area	laser-treated Mature	Peak area
Caffeic acid	15.4	3.86	5115.48	4.41	5844.37	6.44	8534.63	5.64	7474.43	6.08	8057.54
Ferulic acid	27.5	0.03	39.75	0.04	53.01	0.09	119.27	0.06	79.51	0.07	92.76778
Catechin	16.3	1.17	1550.54	1.34	1775.84	1.82	2411.96	1.59	2107.15	1.77	2345.69958
Galic acid	3.1	3.76	4982.95	4.84	6414.22	9.72	12881.46	7.54	9992.41	8.16	10814.07264
p-Coumaric acid	23.3	1.02	1351.75	1.31	1736.08	2.04	2703.51	1.58	2093.90	1.88	2491.47752
kaempferol	56.9	0.44	583.11	0.82	1086.70	1.05	1391.51	0.57	755.39	1.5	1987.881
Chlorogenic acid	16.5	0.11	145.77	0.14	185.53	0.16	212.04	0.12	159.03	0.17	225.29318
Quercetin	28.41	1.54	2040.89	1.98	2624.00	4.28	5672.08	3.32	4399.84	3.51	4651.64154
Luteolin	44.1	0.04	53.01	0.07	92.76	0.14	185.53	0.08	106.02	0.12	159.03048
Apigenin	16.9	0.16	212.04	0.21	278.30	0.44	583.11	0.44	583.11	0.46	609.61684
Naringenin	20.37	0.78	1033.69	1.44	1908.36	2.67	3538.42	1.44	1908.36	2	2650.508
Velutin	12.1	0.01	13.25	0.01	13.25	0.02	26.50	0.02	26.50	0.02	26.50508
Tricin	17.49	0.77	1020.44	1.17	1550.54	2.06	2730.02	1.36	1802.3	1.81	2398.70974
vitexin	30.4	0.51	675.87	0.58	768.64	1.21	1603.55	1.06	1404.76	1	1325.254

Phenolics

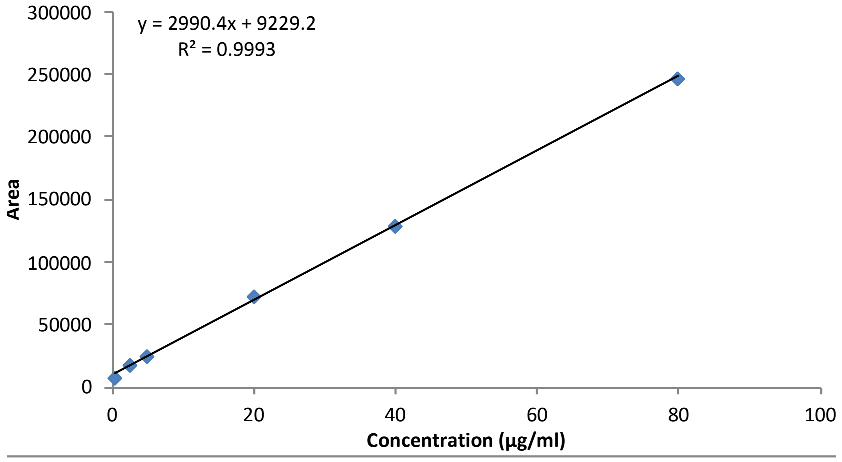

3,5-dichloro-4-hydroxybenzoic acid (Internal standard)

Example of calibration curves of two phenolic standards and chromatogram of the target phenolic standards standards

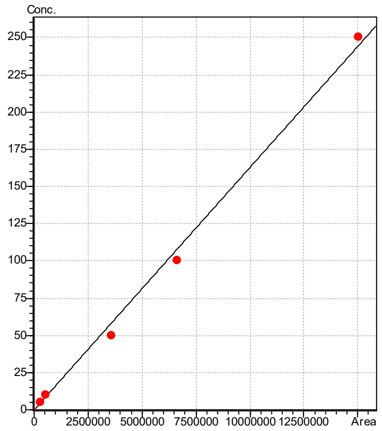

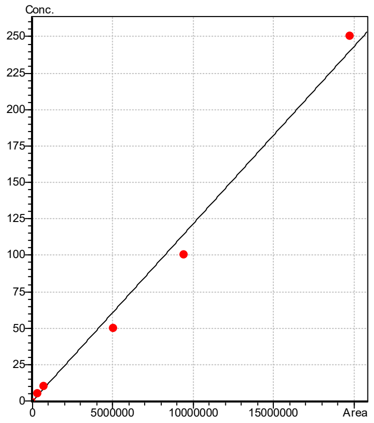

Datafile Name:130618.01.lcd
Sample Name:Y1
Sample ID:Y1

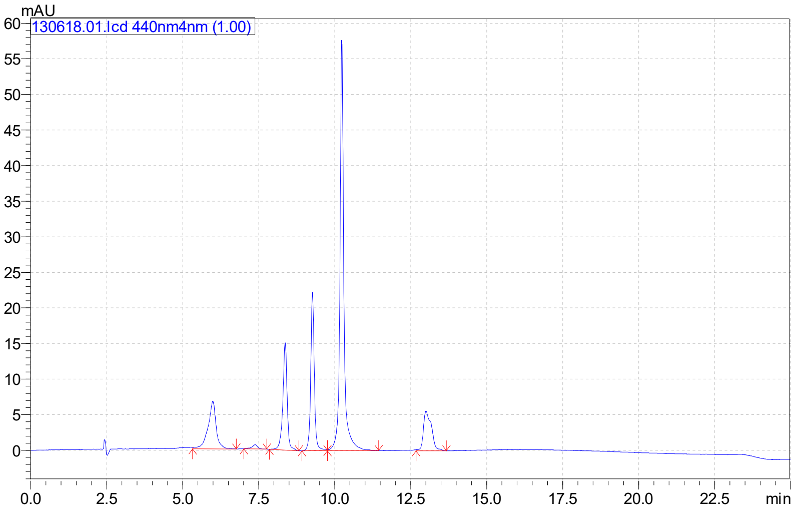

Example of chromatogram of the target pigments standards and sample

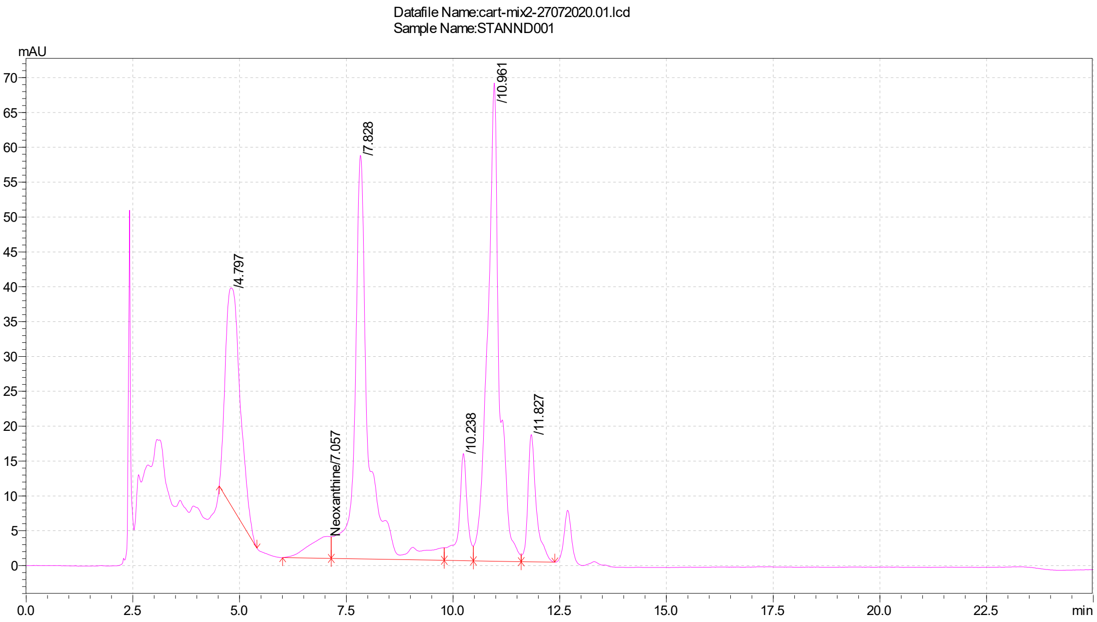

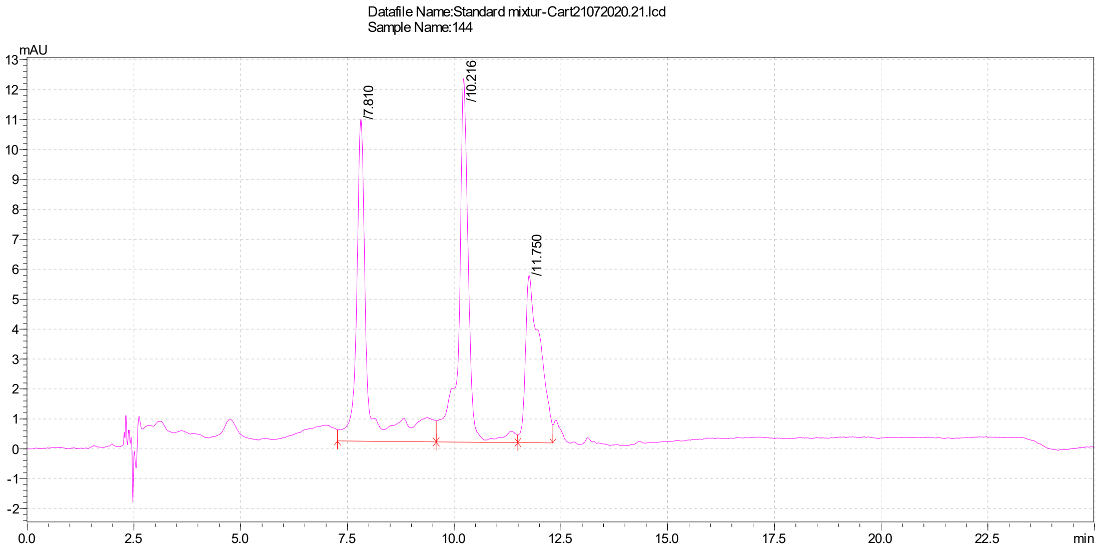

Example of chromatogram of the essential oil profile

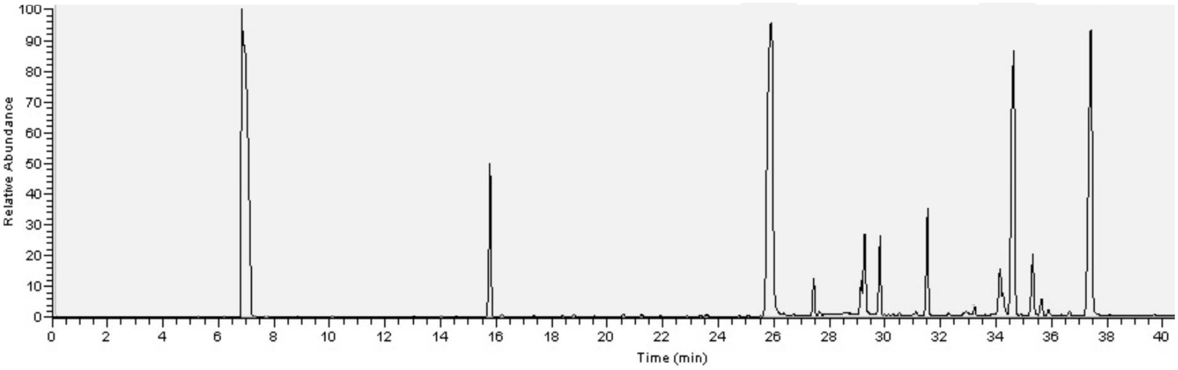
Relative Abundance
Time (min)
